# Extracellular RNA profiles with human age

**DOI:** 10.1111/acel.12785

**Published:** 2018-05-24

**Authors:** Douglas F. Dluzen, Nicole Noren Hooten, Supriyo De, William H. Wood, Yongqing Zhang, Kevin G. Becker, Alan B. Zonderman, Toshiko Tanaka, Luigi Ferrucci, Michele K. Evans

**Affiliations:** ^1^ Department of Biology Morgan State University Baltimore Maryland; ^2^ Laboratory of Epidemiology and Population Science National Institute on Aging National Institutes of Health Baltimore Maryland; ^3^ Laboratory of Genetics and Genomics National Institute on Aging National Institutes of Health Baltimore Maryland; ^4^ Translational Gerontology Branch National Institute on Aging National Institutes of Health Baltimore Maryland

**Keywords:** aging, circular RNA, exRNA, microRNA, ncRNA, women

## Abstract

Circulating extracellular RNAs (exRNAs) are potential biomarkers of disease. We thus hypothesized that age‐related changes in exRNAs can identify age‐related processes. We profiled both large and small RNAs in human serum to investigate changes associated with normal aging. exRNA was sequenced in 13 young (30–32 years) and 10 old (80–85 years) African American women to identify all RNA transcripts present in serum. We identified age‐related differences in several RNA biotypes, including mitochondrial transfer RNAs, mitochondrial ribosomal RNA, and unprocessed pseudogenes. Age‐related differences in unique RNA transcripts were further validated in an expanded cohort. Pathway analysis revealed that EIF2 signaling, oxidative phosphorylation, and mitochondrial dysfunction were among the top pathways shared between young and old. Protein interaction networks revealed distinct clusters of functionally‐related protein‐coding genes in both age groups. These data provide timely and relevant insight into the exRNA repertoire in serum and its change with aging.

## 1 INTRODUCTION

Aging is the predominant risk factor for a myriad of conditions including neurodegenerative, cerebrovascular, cardiovascular, and neoplastic diseases. Significant expansion of the older population highlights the pressing need to discover circulating factors that may serve as potential biomarkers of or therapeutic targets for age‐related diseases, as well as factors associated with resilience and healthy aging. Accumulating evidence suggests that circulating factors play important roles in regulating tissue function and aging. Studies demonstrate that old mice exposed to blood from young mice improved cognitive and muscle function and had less‐evident brain atrophy (Conboy et al., 2005; Katsimpardi et al., 2014; Villeda et al., 2014). The circulating molecules that mediate this effect are yet to be elucidated. The discovery of circulating cell‐free DNA (extracellular DNA; exDNA) and RNA (extracellular RNA; exRNA) in human body fluids, including serum, has sparked great interest in whether these molecules are functional, have regulatory effects, or can be used as markers of disease. For example, exDNA found in maternal serum has led to significant advancements in prenatal testing and diagnosis (Chiu & Lo, 2013; Fan, Blumenfeld, Chitkara, Hudgins & Quake, 2008; Fan et al., 2012; Lo et al., 1997), and for prediction of heart transplant rejection in adults (De Vlaminck et al., 2014; Snyder, Khush, Valantine & Quake, 2011).

ExRNAs have been identified in most bodily fluids including plasma, serum, urine, saliva, and cerebrospinal fluid [for review see (Dluzen, Noren Hooten & Evans, 2017)]. In general, the most well‐studied biotype of exRNA has been microRNAs (miRNAs), but other noncoding RNAs (ncRNAs) are also present in extracellular fluids including Piwi‐interacting RNAs (piRNAs), long noncoding RNAs (lncRNAs), small nuclear RNAs (snRNAs), small nucleolar RNAs (snoRNAs), ribosomal RNAs (rRNAs), transfer RNAs (tRNAs), Y‐RNAs, and circular RNAs (circRNAs) (Amorim et al., 2017; Wei et al., 2017; Yeri et al., 2017). Although not as well‐characterized, fragments or transcripts of messenger RNA (mRNA) have also been identified in extracellular fluid (Amorim et al., 2017; Wei et al., 2017).

Thus far, the majority of exRNA research has used small RNA next generation sequencing approaches to identify only small ncRNAs (Freedman et al., 2016), with the most insightful and provocative findings coming from extracellular, circulating miRNAs. miRNAs are resistant to nucleases due to their small size and the fact that in body fluids, they are likely protected in extracellular evesicles (EVs) or bound to protein or high‐density lipoprotein (HDL) binding partners (Brase, Wuttig, Kuner & Sultmann, 2010; Cortez et al., 2012; Reid, Kirschner & van Zandwijk, 2011; Wagner et al., 2013). This reservoir of miRNAs may transit to other cells and tissues where in some cases they influence gene regulation and may play a role in physiology and pathology (Hergenreider et al., 2012; Wei et al., 2017; Zhang et al., 2010, 2015). Earlier, we found that several circulating miRNAs are lower in abundance as humans age (Noren Hooten et al., 2010, 2013). Furthermore, circulating miRNAs are useful diagnostic markers for the prognosis and response to treatments for a plethora of diseases including cardiovascular disease, diabetes, and cancer (Melman et al., 2015; Schwarzenbach, Nishida, Calin & Pantel, 2014; Willeit et al., 2017).

Here, we sought to identify exRNAs from serum and assess their association with aging. We developed a comprehensive sequencing protocol and analysis strategy to sequence and profile total extracellular RNA (both small and large together) in serum samples as a function of human aging. Our approach not only cataloged and classified the various RNA species in serum from community‐dwelling individuals, but also identified age‐related differences in different types of RNA species. Unlike other studies, we also followed up our sequencing results using real‐time quantitative PCR (RT‐qPCR) to further validate significant age‐related differences. We found significant age‐related differences in several different RNA types, including mRNAs, transcripts expressed from pseudogenes, snoRNAs, miRNAs, and circRNAs. This analysis provides insight into the potential functional roles that exRNAs play in human health, signaling, and disease, and may uncover important diagnostic or therapeutic targets for future studies and interventions in aging and age‐related diseases.

## 2 RESULTS

### 2.1 Summary of the sequencing alignments

**Figure 1:**
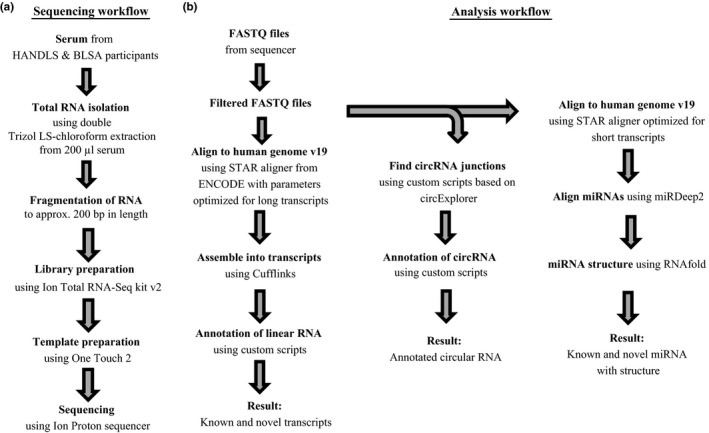
Schematic of sequencing and analysis pipelines. Overview of the study design for sample RNA sequencing (a) and for alignment and identification of linear RNA, miRNA, and circRNA reads (b)

Total, cell‐free extracellular RNA (exRNA) was isolated from human serum from 23 African American females; 13 young participants (30.9 years) from the HANDLS study and 10 old participants from the BLSA study (81.8 years; see Supporting Information Table S1 for complete demographics). Next generation RNA‐seq was performed to capture both long and short RNA transcripts in one comprehensive protocol. There was an average filtered read count of ~7.57 million and ~8.28 million for the young and old individuals, respectively (Supporting Information Table S1). We aligned the sequenced reads to the human genome v19 (hg19) using an analysis pipeline to separately identify linear RNAs, mature miRNAs, or circular RNAs (circRNAs) using available software tools and custom scripts (Figure 1). There was an average of 4.32 million and 4.98 million aligned reads in young and old, respectively.

**Figure 2:**
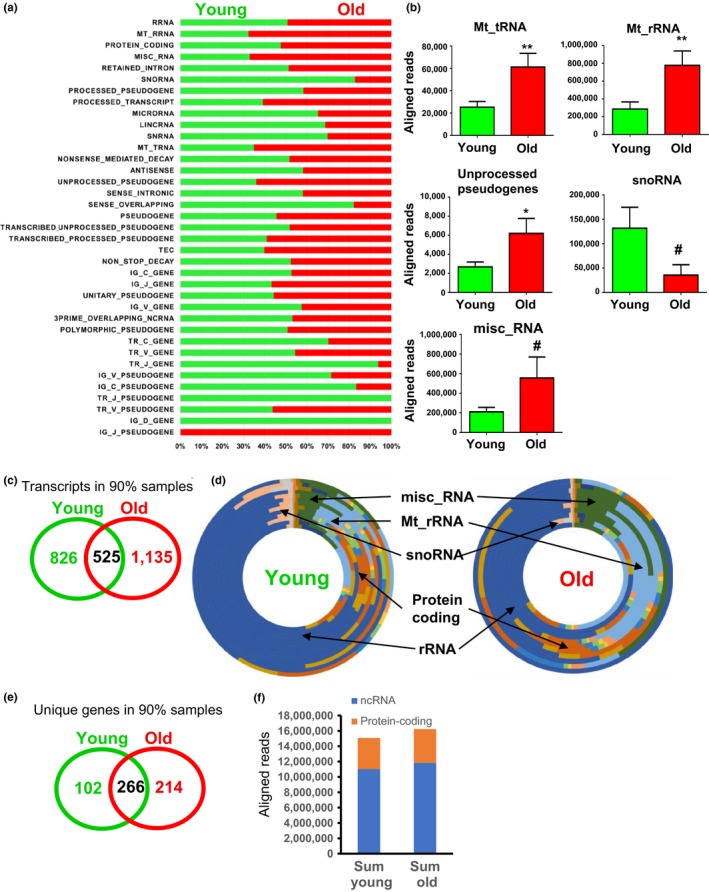
Age‐dependent changes in transcript biotype. (a) Percentage of total aligned reads for RNA from young and old participants by ENSEMBL biotype. Numerical values for each biotype are found in Supporting Information Table S2 (b) Changes in biotype with age are shown. Histograms show the sum of total aligned reads in each age group ± SEM. *p < 0.05, **p < 0.01, or #*p *< 0.09 by Student's *t* test. (c) Venn diagram showing the number of transcripts in 90% of samples in each age group. (d) Variation in transcript biotype is visualized using a donut graph where each layer corresponds to an individual and biotypes of interest are highlighted. (e) Venn diagram showing unique genes in 90% of young/old samples. (f) Comparison of ncRNA vs. protein‐coding RNA composition in exRNA from young and old individuals.

Analysis of aligned read counts identified over 53 and 57 million aligned reads in young and old persons, respectively, classified into 38 Ensembl RNA biotypes (Supporting Information Table S2). We observed variability in the total aligned reads of each biotype between young and old individuals and significant changes with age in the sequenced read counts of mitochondrial rRNAs, mitochondrial tRNAs, and unprocessed pseudogene‐encoded transcripts (Figure 2a,b). Age‐related differences in the levels of snoRNAs and misc_RNAs were also observed but these did not achieve significance (*p *<* *0.09; Figure 2a,b). rRNAs accounted for the majority of exRNA (57.9% young and 64.5% old) (Supporting Information Table S2).

We summarized the number of linear transcripts that were detected in at least one individual through 100% of the individuals in each age group (Supporting Information Table S3). Many unique transcripts were identified in only one individual (115,996 in young and 112,031 in old). Examination of transcripts identified in 90% of each age group found that there were 826 unique to young, 1,135 unique to old individuals and 525 transcripts that were overlapping in both young and old (Figure 2c; Supporting Information Table S3). These patterns suggest that exRNA profiles may be unique to each age group, but also shows the validity of our approach that many exRNA transcripts were identified in the majority of individuals.

We then analyzed the normalized read count (FPKM) for the linear transcripts. We excluded transcripts with ≤1 FPKM, predicted transcripts, and miRNAs from this analysis. We categorized the number of linear transcripts that were detected in at least one individual to 100% of the individuals (Table 1). We also report the number of transcripts that were not only unique to young or old and but also overlapping between the groups and transcripts that showed a significant age‐related change (Table 1). To get a better idea about each individual's exRNA biotypes distribution, we visualized this using a donut graph where each layer represents the distribution of RNA biotype for each individual (Figure 2d). As multiple transcripts (having unique ENSEMBL IDs) can come from the same gene, we wanted to address the number of unique genes in our exRNA profiles. Unique genes were defined by having a single gene symbol. In 90% of our samples, 102 unique genes were identified in young, 214 in old and 266 were shared among the two groups (Figure 2e).

**Table 1:** Number of linear RNA transcripts by FPKM detected in each age range

Sequenced transcripts	>1 FPKM in young	>1 FPKM in old	# overlapping between young and old	# with significant, age‐related changes
Detected in at least 1 sample	21,696	17,049	8,746	–
Detected in 10% of samples	6,640	17,049	4,549	–
Detected in 20% of samples	3,100	4,668	1,968	–
Detected in 30% of samples	1,833	2,094	1,145	–
Detected in 40% of samples	857	1,174	603	–
Detected in 50% of samples	629	772	449	28
Detected in 60% of samples	461	541	331	24
Detected in 70% of samples	203	382	170	17
Detected in 80% of samples	135	249	101	12
Detected in 90% of samples	79	142	55	9
Detected in 100% of samples	36	50	21	2

Recent analysis of exRNA secreted in extracellular vesicles (EVs) from glioblastoma cells in vitro indicates that ncRNA composes the majority of exRNA species compared with mRNA (Wei et al., 2017). To establish whether this trend is similar for exRNA in serum, we categorized the transcript biotypes into either ncRNA or protein‐coding RNA using ENSEMBL classifications (Supporting Information Table S2). We removed Mt_rRNA, rRNA and to be experimentally confirmed (TEC) biotypes. The total aligned reads for each biotype were used for our comparison. ncRNAs were the most abundant RNA class in both young and old individuals compared with mRNA (Figure 2f).

**Figure 3:**
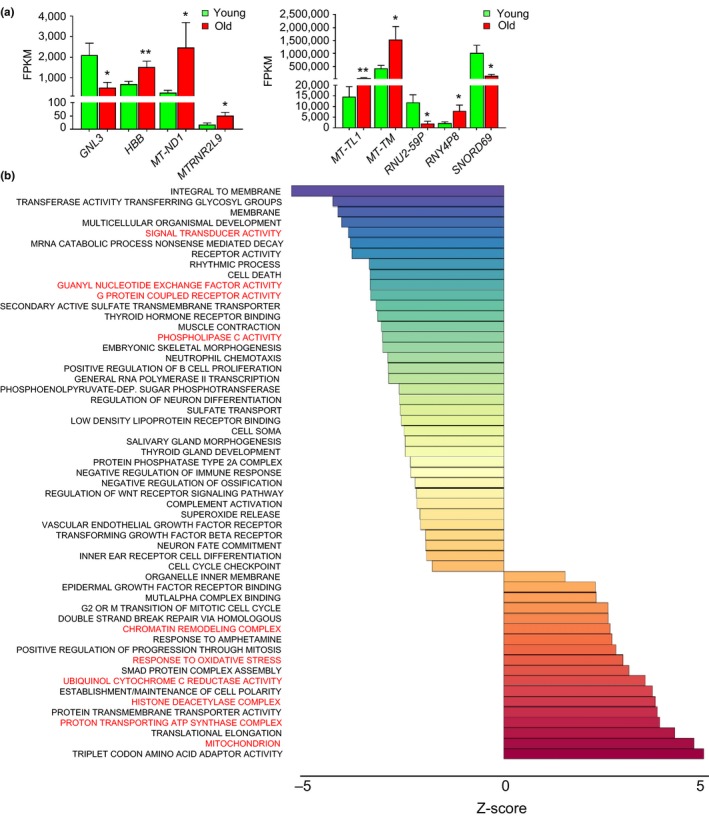
Gene and pathway changes with age. (a) FPKM values for genes that were significantly different with age are shown. Histogram represents the mean + SEM. *p < 0.05 and **p < 0.01 and by Student's *t* test. (b) DESeq2 gene set of differential genes between young and old (Supporting Information Table S4) was imputed into PAGE to analyze the Gene Ontology gene sets. The top significant GO terms are shown and all the associated data are in Supporting Information Table S5

We initially compared FPKM differences in linear transcripts and found several different transcripts that were differentially expressed with age (Figure 3a). Given the large dynamic range in our dataset, we chose to use the DESeq2 algorithm in BRB‐Arraytools (Love, Huber & Anders, 2014) as this is a more suitable computational tool for comparative analysis in our dataset, particularly because it can assess differential‐gene expression in datasets with large ranges. DESeq2 identified 1,154 genes significantly different between young and old (Supporting Information Table S4). We imputed our DESeq2 gene set into PAGE analysis to identify enriched gene ontology (GO) gene sets. GO analysis between young and old identified significant enrichment in older individuals in genes related to the mitochondrion, response to oxidative stress, and chromatin remodeling, among others. Top GO terms with significantly decreased enrichment in old include various signaling pathways including signal transducer activity, receptor activity, and others (Figure 3b). A complete list of all GO terms and those broken down by biological process, molecular function, and cellular component can be found in Supporting Information Table S5.

### 2.2 Validation of age‐related changes in exRNA

**Figure 4:**
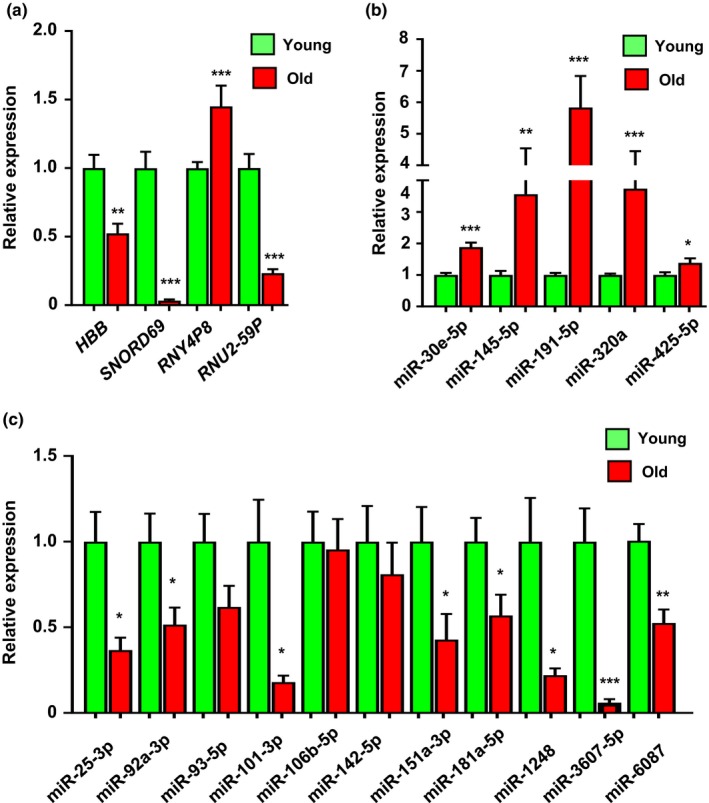
ExRNA changes with age. (a) RNA was isolated from serum from an expanded cohort of young (*n* =39; 31.09 ± 0.97 years) and old individuals (*n* = 20; 81.8 ± 1.26 years.). RNA was reverse transcribed as described in the methods to examine linear RNA transcript of different biotypes (a) and miRNA levels (b and c) by RT‐qPCR. Histograms show the relative expression in each age group ± SEM. *p < 0.05, **p < 0.01, ***p < 0.001 by Student's *t* test

To assess the quality of our sequencing analysis, we set out to validate various transcripts in serum by RT‐qPCR analysis using an expanded young (*n* = 39; 31.09 ± 0.97 years) and old (*n* = 20; 81.8 ± 1.26 years) cohort from HANDLS and BLSA, respectively. We chose to validate the expression of different RNA biotypes and selected several transcripts that showed the strongest age‐dependent changes in our sequencing analysis in either the FPKM or DESeq2 analyses, including *SNORD69* which was found along with *MT‐TL1* and *MT‐ND1* to be significantly changed with age in both analyses. In addition, we examined the sequencing coverage for each transcript chosen for validation (Supporting Information Figures S1–S4). Although the coverage varied per individual and per gene, in some genes, we detected sequences across the majority of the exons, but in others, we did not (Supporting Information Figures S1–S4). The coverage information was used for gene‐specific primer design (sequences in Supporting Information Table S2). We found significant age‐related changes in a coding transcript (*HBB* mRNA), a small nucleolar RNA (*SNORD69*), a pseudogene transcript (*RNY4P8*), and a small nuclear pseudogene transcript (*RNU2‐59P*) (Figure 4a). These data indicate we can identify and validate age‐related changes in exRNAs.

In our sequencing pipeline (Figure 1b), we identified a total of 545 circulating miRNAs. We used DESeq2 to analyze differentially expressed miRNAs between young and old and identified 12 miRNAs that were significantly different between age groups. Recently, we analyzed existing studies in the literature to identify a number of circulating miRNAs that change with age in multiple studies (Dluzen et al., 2017). Here, we chose these miRNAs to further validate in this independent cohort (miR‐25‐3p, miR‐92a‐3p, miR‐93‐5p, miR‐101‐3p, miR‐106b‐5p, miR‐142‐5p, miR‐151a‐3p, and miR‐181a‐5p). With the exception of miR‐106b‐5p and miR‐142‐5p, all the other miRNAs we validated were found to be significantly downregulated with age, including miR‐151a‐5p, miR‐181a‐5p, and miR‐1248, which we previously found to be decreased in older individuals in a different aging cohort (Noren Hooten et al., 2013). We also validated new age‐related miRNAs identified in our current sequencing analysis and identified several new aging‐related miRNAs including miR‐30e‐5p, miR‐145‐5p, miR‐191‐5p, miR‐320a, miR‐3607‐5p, miR‐425‐5p, and miR‐6087 (Figure 4b,c).

### 2.3 ExRNA pathway analysis

We wanted to better understand the biological pathways that are represented by the genes we identified. We used the STRING database to generate a protein–protein interaction network of the genes detected in the circulation of young and old individuals. STRING assembles associations of either known or predicted protein–protein interactions to build a genome‐wide functional network (Szklarczyk et al., 2017). Each node represents a different exRNA, the colors identify clusters, and the lines signify the different functional (direct or indirect) relationship. Several different clusters were identified containing genes encoding mitochondrial proteins, ribosomal proteins, and histones/transcription factors (Supporting Information Figures S5 and S6). Similar clusters were observed in both young and old individuals, yet the clusters were not as well‐organized in the old as in the young (Supporting Information Figures S5 and S6). These networks suggest that the exRNAs we identified are related functionally and not just a random assortment of RNAs.

To gain additional information about the pathways that may be regulated by these genes, we used Ingenuity Pathway Analysis to assess the Top Canonical Pathways and Molecular and Cellular functions that are regulated by these exRNAs (Table 2). Overlapping pathways were identified in both young and old including EIF2 signaling, oxidative phosphorylation, mTOR signaling, and mitochondrial dysfunction (Table 2). Some pathways were unique to an age group. For example, Systemic Lupus Erythematosus Signaling was only identified in old, which is interesting considering that Lupus is an autoimmune disease that is more prevalent in African American women.

**Table 2:** Pathway analysis for exRNA from young and old

Young	Old
**Top canonical pathways**	**Top canonical pathways**
EIF2 signaling	EIF2 signaling
Oxidative phosphorylation	Systemic lupus erythematosus signaling
Mitochondrial dysfunction	mTOR signaling
mTOR signaling	Oxidative phosphorylation
Protein kinase A signaling	Regulation of eIF4 and p70S6K signaling
Regulation of eIF4 and p70S6K signaling	Mitochondrial dysfunction
**Top diseases and biological functions**	**Top diseases and biological functions**
Cancer	Cancer
Hematological disease	Hematological disease
Immunological disease	Organismal Injury and abnormalities
Organismal Injury and abnormalities	Immunological disease
Tumor morphology	Tumor morphology
**Molecular and cellular functions**	**Molecular and cellular functions**
Cell death and survival	Cell death and survival
Protein synthesis	Free radical scavenging
Cell cycle	Cellular movement
DNA replication, recombination, and repair	Protein synthesis
Cell‐to‐cell signaling and interaction	Carbohydrate metabolism

We also analyzed the top molecular and cellular functions predicted to be regulated by these exRNAs (Table 2). Several of these pathways are considered as various hallmarks of aging including protein synthesis, cell cycle, DNA replication, recombination and repair, and cell‐to‐cell signaling and interaction, which were among the top functions represented in our younger cohort (Lopez‐Otin, Blasco, Partridge, Serrano & Kroemer, 2013). In old individuals, free radical signaling and carbohydrate metabolism, which are also dysregulated during the aging process, were also among the top functions (Lopez‐Otin et al., 2013).

### 2.4 circRNAs are present in serum and change with age

**Figure 5:**
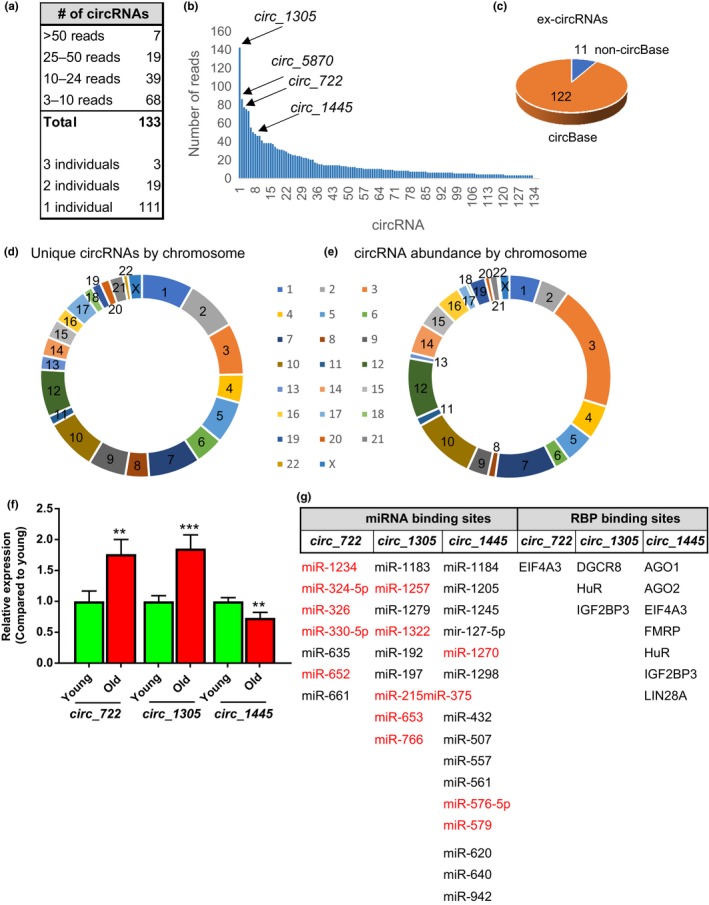
circRNAs are present in serum and change with age. (a, b) Analysis of number of circRNAs and abundance from our sequencing analysis. circRNAs that were validated by RT‐qPCR are indicated. (c) The number of extracellular circRNAs (ex‐circRNAs) that are found in circBase are indicated. (d) ex‐circRNA abundance by chromosome. (e) Unique ex‐circRNAs by chromosomes are indicated. (f) miRNA and RBP binding sites in the specified circRNAs are listed and serum miRNAs present in our cohort are indicated in red. (G) circRNA expression from our validation cohort was analyzed by RT‐qPCR. ***p < 0.001 and **p ≤ 0.01 by Student's *t* test

In addition to examining linear transcripts and miRNAs, we used scripts to identify exon‐junction anomalies indicative of circRNAs. We next ascertained and annotated the potential circRNAs using custom scripts based on circExplorer and found 133 circRNAs with >3 reads total in serum (Figure 5a,b; Supporting Information Table S6). Of these 133 circRNAs, 122 of them were present in the circRNA database, circBase (Figure 5c) (Hudson, Stark, Fast, Russell & Rader, 2015). The 11 circRNAs we identified that are not annotated in circBase are *hg19_circ_chr12_46764960_46765162_R, hg19_circ_chr5_131034609_131055110_R, hg19_circ_chr9_100823063_100840629_F, hg19_circ_chr7_65945380_65953025_R, hg19_circ_chr10_12272946_12280484_F, hg19_circ_chr8_82583173_82593819_R, hg19_circ_chr16_69718789_69728142_F, hg19_circ_chr9_139341423_139341678_R, hg19_circ_chr17_29164223_29167799_F, hg19_circ_chr21_30434648_30434736_R,* and *hg19_circ_chr5_169108747_169122942_F* (Supporting Information Table S6).

The majority of circRNAs were low in abundance; however, several were detected at a higher number of reads (Figure 5a,b). circRNAs were derived from all chromosomes with chromosomes 1, 2, 3, 7, and 10 containing the greatest number of unique circRNA transcripts (Figure 5d). The most abundant circRNAs were from chromosomes 3, 10, and 12 (Figure 5e).

As circRNAs have not been extensively investigated from serum or plasma, we chose to further enhance our computational approach by validating the presence of circRNAs using RT‐qPCR analysis. We chose several circRNAs to validate in our larger cohort of young and old individuals. These circRNAs were the most abundant and/or were also present in ≥2 individuals in our sequencing. Divergent primers for each circRNA were designed using CircInteractome (Dudekula et al., 2016). Each primer set was validated initially by examining RNA isolated from PBMCs, HUVECs, and serum. Amplification plots, dissociation curves, and analysis of RT‐qPCR products showed that each primer set amplified a single RT‐qPCR product indicative of amplification of a single circRNA (Supporting Information Figures S7 and S8). Products from the RT‐qPCR reactions were analyzed by gel electrophoresis to further visualize that a single RT‐qPCR product was amplified (Supporting Information Figures S7 and S8). Intriguingly, the highly abundant *hsa‐circ_0001305 (circ_1305)* and *hsa‐circ_0001445 (circ_1445)* were present in serum, but were not detectable in PBMCs or HUVECs (Supporting Information Figure S7). *hsa‐circ_0000722* (*circ_722*) was present in serum and PBMCs but at lower levels in HUVECs (Supporting Information Figure S7). We designed multiple primer sets to analyze *hsa‐circ_0005870* expression, but we were unable to amplify this circRNA from PBMCs, HUVECs, or serum (data not shown). Therefore, we did not analyze this circRNA in our expanded cohort of individuals.

Examination of circRNAs in our expanded young and old cohort revealed that circRNAs *circ_1305* and *circ_722* were significantly higher and *circ_1445* was significantly lower in old individuals compared with young (Figure 5g; Supporting Information Figure S8). Analysis of RT‐qPCR products also showed a single band indicative of amplification of a single circRNA (Supporting Information Figure S8). circRNAs contain binding sites for both miRNAs and RNA‐binding proteins, and here, we found that these extracellular circRNAs (ex‐circRNAs) contain predicted binding sites for both miRNA and RBPs (Figure 5f). Several of the predicted miRNAs (highlighted in red in Figure 5f) were also present in serum in our sequencing analysis. The transcripts encoding the RBPs that were predicted to bind were not present in serum in our cohort. Together, not only did our sequencing analysis detect the presence of circRNAs in serum, but we also validated that three circRNAs are significantly changed with age.

## 3 DISCUSSION

Here, we have cataloged and classified the various serum exRNA species in young and old individuals. Our unique approach also identified age‐related differences in different types of RNA species. It is interesting that we found higher serum levels of both mitochondrial tRNAs and mitochondrial rRNAs with age. This is consistent with data in the literature that mitochondrial function is progressively impaired with aging in animal models as well as in humans and is a hallmark of aging (Lopez‐Otin et al., 2013). Our network analysis identified a cluster of mRNAs encoding mitochondrial proteins (Supporting Information Figures S5 and S6). Furthermore, our pathway analysis identified mitochondrial dysfunction and oxidative phosphorylation among the top canonical pathways. Cells have been found to shed lysosome‐like vesicles and entire mitochondria into EVs through a mitophagy‐dependent mechanism (Phinney et al., 2015). In fact, mesenchymal stem cells use this mechanism to release mitochondria in EVs in response to oxidative stress (Phinney et al., 2015). Here, perhaps the higher levels of mitochondrial RNAs in serum with age may be a consequence of mitochondrial dysfunction and higher levels of oxidative stress that occur with age.

We also observed that many different RNA biotypes were equally distributed among exRNA from young and old individuals, and many transcripts were detected in both young and old individuals. These data show the validity of our sequencing approach and suggest that a relative consistency among age groups in the exRNA profiles. In both young and old women, ncRNAs were more abundant than mRNAs (Figure 2). These data are consistent with a recent report comparing RNA isolated from cells and EVs from glioma stem cells (Wei et al., 2017). However, we also observed age‐related differences among snoRNAs. Although the difference was not significant overall for this biotype, we did follow up in our validation studies and found significant differences in a specific snoRNA, *SNORD69*, which was found to be differentially expressed using either FPKM or DESeq2 analysis (Figures 3 and 4). A recent study has also identified snoRNAs in plasma (Freedman et al., 2016); however, differences in nomenclature impair the ability to cross‐compare the studies. Nevertheless, in this study, several snoRNAs were found to be decreased in plasma of individuals >66 years (Freedman et al., 2016), which is consistent with our snoRNA data.

A limited number of papers have examined whether circRNAs may be present in serum, mostly using computational methods (Amorim et al., 2017; Gu et al., 2017; Wei et al., 2017). Here, we have used a combination approach and identified 133 extracellular circRNAs (ex‐circRNAs) and further validated age‐related changes in three different ex‐circRNAs: *circ_1305*,* circ_722,* and *circ_1445*. Emerging data suggest that circRNAs can function to sponge miRNAs and RBPs (Panda, Grammatikakis, Munk, Gorospe & Abdelmohsen, 2017). Here, we have identified several serum miRNAs that are predicted to bind to the ex‐circRNAs and also with several RBPs. It is interesting to speculate that these ex‐circRNAs may be acting as decoys to bind to various miRNAs, RBPs, or perhaps other ncRNAs in the circulation or may act to illicit an immune response to potential infections (Chen et al., 2017). Future work lies in further delineating the spectrum of functions of ex‐circRNAs.

In addition to validating circRNAs by RT‐qPCR, we also validated the levels of various serum miRNAs. We chose to validate miRNAs that we identified in our DESeq2 analysis to be differentially expressed with age. For a cross‐study comparison, we chose many miRNAs that previously our group and others found to be changed with age (Dluzen et al., 2017; Noren Hooten et al., 2013). Many of these miRNAs significantly changed with age in serum in our study. These data will be valuable to the field as many serum miRNAs have potential use as clinical tools.

In this study, we developed a protocol to sequence both small and large RNAs in one sequencing reaction, which will enable researchers to save on cost and time. We used this protocol to examine exRNA species from a readily available biofluid, serum, to identify exRNA species that are altered with age. Therefore, this approach is readily amenable for biomedical researchers to use as a platform for the development of clinical tools based on circulating exRNA. At present, there are limited studies examining exRNA, both small and large RNAs, in the context of aging. Our findings are a start at building a bridge over this knowledge gap. In addition, our study validates a recently published approach that incorporates RNA fractionation as a method to capture both short and long RNAs in one sequencing reaction (Amorim et al., 2017), with small differences in the method to fractionate RNA.

Of note, the approach we have used does not distinguish between exRNA associated with EVs, RBPs, HDL or those which are nonvesicular. A recent cross‐comparison analysis of the different fractions suggests that exRNA species and abundance do vary between exosomes, microvesicles, and the nonvesicular fractions (Wei et al., 2017). This extensive study was performed from glioma stem cells grown in vitro, which allows for large scale experiments. Here, we are limited by sample volume and availability. Nevertheless, as larger datasets of exRNAs emerge, we can gain important knowledge about the landscape of exRNA in a variety of different biofluids and from cells. In summary, our comprehensive protocol to sequence small and large RNA species in one reaction will enable the systematic identification of exRNAs associated with functional decline and diseases of aging. Furthermore, our data provide important steps to classifying and cataloging the repertoire of exRNAs in the circulation. This insight will aid in identifying whether exRNAs are suitable candidates for future diagnostics or therapeutics.

## 4 EXPERIMENTAL PROCEDURES

### 4.1 Study participants

Participants were chosen from either the Healthy Aging in Neighborhoods of Diversity across the Life Span (HANDLS) study or the Baltimore Longitudinal Study of Aging (BLSA), both conducted by the National Institute on Aging Intramural Research Program (NIA IRP), National Institutes of Health (NIH; Supporting Information Table S1). HANDLS is a longitudinal, epidemiological study based in Baltimore, MD that examines the relationship between race and socioeconomic status on aging and age‐associated health disparities. HANDLS participants are African American (AA) and white men and women residents of Baltimore, Maryland who were between the ages of 30 and 64 at baseline (Evans et al., 2010). The BLSA focuses on healthy aging, as well as age‐associated health issues, by examining a longitudinal cohort of adults (Shock, 1984). HANDLS and BLSA are ongoing studies that have been approved by the Institutional Review Board of the National Institute of Environmental Health Sciences (NIEHS), NIH. All participants provided written informed consent.

For this study, we chose young AA females (30–32 years) from HANDLS and older AA females (80–85 years) from BLSA. (see Supporting Information Table S1). The expanded cohort for validation studies consisted of young (*n* = 39; 31.09 ± 0.97 years) and old (*n* = 20; 81.8 ± 1.26 years) AA females. Fasting blood samples were obtained from individuals in vials with no additives, centrifuged, and serum was collected, aliquoted, and immediately stored at −80°C. Samples from both studies were processed in the same core laboratory at the NIA IRP and individuals were chosen from similar time frames of visits. We excluded participants with documented Hepatitis B, Hepatitis C, or HIV infection, or with a history of cancer, lupus, or irritable bowel disease.

### 4.2 Isolation and preparation of RNA from serum

Total RNA was isolated from 200 μl of serum from 13 young (30.9 ± 0.60 years) HANDLS participants and 10 old (81.8 ± 1.87 years) BLSA participants using TRIzol LS (Life Technologies, Waltham, MA) according to the manufacturer's instructions and with the addition of a second phenol/chloroform extraction prior to RNA precipitation as previously described (Noren Hooten et al., 2013). RNA samples were resuspended in 20 μl of RNAse‐free water and frozen at −80°C until further use. RNA quality was assessed using an Agilent BioAnalyzer (Agilent Technologies, Santa Clara, CA). Total RNA was fragmented to <200 bp using the Bioruptor NGS (Diagenode, Denville, NJ). In brief, RNA was diluted in RNase‐free water to a final volume of 100 μl and subjected to bioruption for 30 min (Power: H position) in an ice‐cold DI water bath using 30”/30” ON/OFF cycles with cold water bath replacement every 7.5 min. Sample RNA was then dry vacuumed to 20 μl and tested for fragmentation using the Agilent BioAnalyzer Small RNA chip.

### 4.3 Library preparation and next generation sequencing

Total RNA isolated as described above was prepared for sequencing using the Ion Total RNA‐seq Kit v2 (Life Technologies) with multiplexing according to the manufacturer's instructions. The Life Technologies Ion RNA Adaptors were added to the fragmented RNA at 65°C for 10 min and then allowed to hybridize at 30°C for 5 min. A ligation reaction was carried out at 30°C for 1 hr. The Life Technologies reverse transcription primer was incubated with the ligated samples at 70°C followed by snap‐cooling on ice for hybridization. A reverse transcription reaction was performed using Superscript III at 42°C for 30 min. Clean‐up of the cDNA was performed using the supplied magnetic bead clean‐up module and 100% ethanol. Samples were eluted from the beads using 37°C nuclease‐free water. Amplification and addition of sample‐specific indexes were performed using 16 cycles of PCR to enrich the samples for fragments that contained adaptors on both ends, followed by another magnetic bead clean‐up. These enriched samples were tested using the Agilent BioAnalyzer High Sensitivity DNA chips. Barcoded samples were pooled, and each library template was clonally amplified on Ion Sphere™ Particles in the Ion One Touch II for sequencing on the Ion Proton™ System.

### 4.4 Sequence analysis and database comparisons

Total RNA‐seq analysis was performed to identify long linear RNA, circular RNA, and microRNA using different software tools and in‐house custom scripts (see Figure 1). In short, raw fastq files were obtained using Life Technologies Torrent Software Suite and cleaned using the cutadapt program. Samples with sequenced read counts <1,000,000 were excluded from the analysis.

The sequences were aligned against the human genome version 19 using STAR from ENCODE and assembled using Cufflinks against the Ensembl human transcript annotation v72. Transcript and gene read counts were obtained using subread featureCounts software. The “fusion reads” were parsed and circRNAs were identified from these “fusion reads” using CircExplorer (v1.1). For circRNA analysis, any circRNA detected that contained <3 reads in all individuals was excluded. miRNAs were identified using mirDeep2 software (Friedlander et al., 2008). Differential miRNA and gene expression between young and old was determined using DESeq2 (Love et al., 2014) algorithm in BRB‐Arraytools. BRB‐ArrayTools was developed by Dr. Richard Simon and the BRB‐ArrayTools Development Team (https://brb.nci.nih.gov/BRB-ArrayTools/). All data were annotated using custom scripts. Data are available at GEO (Accession Number: GSE112289).

### 4.5 Transcript validation

Total RNA was isolated from 50 μl of human serum using the same protocol described above and RNA was resuspended in 20 μl RNAse‐free water. For linear RNA transcripts, 10 μl of RNA was transcribed into cDNA using random hexamers and Superscript II Reverse Transcriptase (Invitrogen). For miRNA, the remaining 10 μl was transcribed into cDNA using the QuantiMiR RT Kit (Systems Biosciences, Mountain View, CA). Reactions were performed with 2× SYBR Green Master Mix and gene‐specific primers (Supporting Information Table S7). Reverse transcription and quantitative real‐time PCR (RT‐qPCR) reactions were performed on a 7900HT Fast Real‐Time PCR System or 7500 Real‐Time PCR System according to the manufacturer's protocols (Life Technologies). Linear RNA transcripts were normalized to the average of *RNA5SP226, RN7SL5P, RNA5SP348*, and *RNY4P10* and miRNAs were normalized to the average of miR‐21‐5p, miR‐451a, and miR‐126‐5p. Normalization transcripts were chosen as they had the least interindividual variability in expression in our sequencing and validation cohorts. Transcript expression levels were examined for Gaussian distribution by measuring kurtosis and skewness and outliers for each transcript were excluded from the analysis using Grubb's test with an alpha of 0.05.

Divergent primers for circRNAs were designed using the CircInteractome web tool (https://circinteractome.nia.nih.gov/Divergent_Primers/divergent_primers.html) (Dudekula et al., 2016). cDNA used to validate linear transcripts (see above) was used for RT‐qPCR reactions. Primer pairs are included in Supporting Information Table S7 and were validated using RNA isolated from PBMCs, human umbilical vein endothelial cells (HUVECs), or control serum. circRNAs were normalized as described above for linear transcripts.

### 4.6 Pathways analysis

exRNAs identified from young and old individuals were imported into Ingenuity Pathway Analysis (IPA; Ingenuity Systems, Redwood City, CA) to identify top **c**anonical pathways, top diseases, biological functions, and molecular and cellular functions. Function or Pathway significance is determined using the Fisher's exact test. exRNAs were also imported into the STRING db (Szklarczyk et al., 2017). Disconnected nodes were removed and clustering was performed using k‐means clustering with default settings throughout. PAGE analysis of GO terms using the genes from the DESeq2 analysis was performed as previously described (Noren Hooten et al., 2013).

### 4.7 Statistics

The two‐tailed Student's t test was used when comparing two groups, unless otherwise noted. A *p*‐value < 0.05 was considered statistically significant.

## CONFLICT OF INTEREST

The authors declare that they have no conflict of interests.

## AUTHORS' CONTRIBUTION

DFD, NNH, KGB, TT, ABZ, and MKE conceived and designed the study. DFD and NNH executed the experiments. WW, SD, and KGB completed the sequencing and SD performed the sequencing analysis. YZ did the GO analysis. DFD, NNH, SD, and KGB analyzed the data. MKE and ABZ are co‐principal investigators for HANDLS. LF is principal investigator for BLSA. DFD and NNH wrote the manuscript. All authors reviewed the manuscript.
