# Supplementary material for: Extracellular RNA profiles with human age
Source: Aging Cell. 2018 May 24;17(4):e12785. doi: 10.1111/acel.12785 (PMC6052399; doi:10.1111/acel.12785)

SUPPORTING INFORMATION

Extracellular RNA profiles with human age

Douglas F. Dluzen[1*], Nicole Noren Hooten[2*], Supriyo De[3], William H. Wood III[3], Kevin G. Becker[3], Alan B. Zonderman[2], Toshiko Tanaka[4], Luigi Ferrucci[4], and Michele K. Evans[2]

[1]Department of Biology, Morgan State University, Baltimore, MD 21251, [2]Laboratory of Epidemiology and Population Science, [3]Laboratory of Genetics and Genomics; [4]Translational Gerontology Branch; National Institute on Aging, National Institutes of Health, Baltimore, MD 21224

*These authors contributed equally to this work.

Table S1: Demographics and Sequenced Reads for Sequencing Cohort

Characteristic	AA Young Females (n=13)	AA Old Females (n=10)
Study	HANDLS	BLSA
Age, y	30.9 ± 0.60	81.8 ± 1.87
Filtered Reads (millions ± S.D.)	7.57 ± 5.06	8.28 ± 6.85
Minimum (millions)	0.523	1.54
Maximum (millions)	16.4	25.3
Aligned Reads (millions ± S.D.)	4.32 ± 3.57	4.98 ± 5.82
Total cholesterol, mg/dL	170 ± 38.6	230 ± 44.0*
HDL, mg/dL	47.1 ± 12.4	76.6 ± 25.0*
LDL, mg/dL	N/A	135.4 ± 44.7*
Triglycerides, mg/dL	84.5 ± 37.3	81.8 ± 48.0*
Fasting, N (% Total)	13 (100)	N/A
Myocardial Infarction, n (% Total)	0 (0.00)	0 (0.00)
Congestive heart failure, n (% Total)	1 (7.69)	0 (0.00)
Stroke, n (% Total)	0 (0.00)	0 (0.00)
Dx Coronary artery disease, n (% Total)	0 (0.00)	1 (10.0)
Dx dementia, n (% Total)	0 (0.00)	0 (0.00
Dx Alzheimer’s, n (% Total)	0 (0.00)	0 (0.00)
Dx cancer, n (% Total)	0 (0.00)	1 (10.0)
Dx diabetes, n (% Total)	1 (7.69)	0 (0.00)
Dx pre-diabetes, n (% Total)	2 (15.4)	0 (0.00)
Dx Hypertension, n (% Total)	3 (23.1)	6 (60.0)
Below Poverty, N (% Total)	7 (53.8)	N/A
Smoking status, Current N (% Total)	5 (38.5)	0 (0.00)
Smoking status, Former N (% Total)	1 (7.69)	4 (40.0)

Table S2: Number of aligned reads of linear and miRNA RNA transcript biotypes detected in each age range

Transcript Biotype	RNA class	Sum Young	Sum Old	P-value
Mt_rRNA	ncRNA	3719871	7778187	0.01
Mt_tRNA	ncRNA	328656	612392	0.01
unprocessed_pseudogene	ncRNA	34820	61926	0.03
snoRNA	ncRNA	1712520	355178	0.08
misc_RNA	ncRNA	2733063	5558488	0.09
TEC	Protein-coding/Needs-Validation	2248	3393	0.1
sense_overlapping	ncRNA	9304	2014	0.14
protein_coding	Protein-coding	3718226	4098050	0.15
lincRNA	ncRNA	550417	251621	0.16
processed_transcript	ncRNA	751121	1166680	0.18
transcribed_processed_pseudogene	ncRNA	3363	4837	0.2
IG_J_pseudogene	ncRNA	0	1	0.26
TR_J_gene	Protein-coding	119	8	0.29
unitary_pseudogene	ncRNA	424	535	0.29
IG_V_pseudogene	ncRNA	98	39	0.34
pseudogene	ncRNA	7034	8410	0.36
snRNA	ncRNA	530959	230363	0.36
IG_C_pseudogene	ncRNA	40	8	0.38
IG_D_gene	Protein-coding	1	0	0.39
TR_J_pseudogene	ncRNA	33	0	0.39
transcribed_unprocessed_pseudogene	ncRNA	4192	3891	0.39
TR_C_gene	Protein-coding	268	114	0.42
nonsense_mediated_decay	Protein-coding	325601	304733	0.44
IG_J_gene	Protein-coding	686	900	0.46
TR_V_pseudogene	ncRNA	7	9	0.51
microRNA	ncRNA	634586	336719	0.58
polymorphic_pseudogene	Protein-coding	342	330	0.6
retained_intron	ncRNA	2438306	2311309	0.66
rRNA	ncRNA	34239600	33073653	0.67
non_stop_decay	Protein-coding	1642	1492	0.75
3prime_overlapping_ncrna	ncRNA	357	315	0.81
antisense	ncRNA	226004	162434	0.81
IG_C_gene	Protein-coding	795	713	0.81
processed_pseudogene	ncRNA	1042443	744214	0.81
sense_intronic	ncRNA	13525	9749	0.85
TR_V_gene	Protein-coding	141	118	0.93
IG_V_gene	Protein-coding	423	313	0.95

Table S3: Number of linear RNA transcripts with at least one sequenced read detected in each age range

	**Young**	**Old**	**# Overlapping between Young & Old**
Detected in at least 1 sample	115,996	112,031	88,997
Detected in 10% of samples	73,405	112,031	64,615
Detected in 20% of samples	47,968	65,128	38,144
Detected in 30% of samples	31,803	37,929	22,885
Detected in 40% of samples	13,871	21,754	10,527
Detected in 50% of samples	8,783	12,150	6,127
Detected in 60% of samples	5,679	6,949	3,595
Detected in 70% of samples	2,143	3,959	1,580
Detected in 80% of samples	1,399	2,147	941
Detected in 90% of samples	826	1,135	525
Detected in 100% of samples	451	618	332

Table S7: Primer Sequences for PCR Validation

Gene	Forward Primer	Reverse Primer
miR-101-3p	TACAGTACTGTGATAACTGAA	
miR-106b-5p	TAAAGTGCTGACAGTGCAGAT	
miR-1248	ACCTTCTTGTATAAGCACTGTGCTAAA	
miR-126-5p	CATTATTACTTTTGGTACGCG	
miR-142-5p	CATAAAGTAGAAAGCACTACT	
miR-145-5p	GTCCAGTTTTCCCAGGAATCCCT	
miR-151a-3p	CTAGACTGAAGCTCCTTGAGC	
miR-181a-1-5p	AACATTCAACGCTGTCGGTGAGT	
miR-191-5p	CAACGGAATCCCAAAAGCAGCTG	
miR-21-5p	TAGCTTATCAGACTGATGTTGA	
miR-25-3p	CATTGCACTTGTCTCGGTCTGA	
miR-30e-5p	TGTAAACATCCTTGACTGGAAG	
miR-320a	AAAAGCTGGGTTGAGAGGGCGA	
miR-3607-5p	GCATGTGATGAAGCAAATCAGT	
miR-451a	AAACCGTTACCATTACTGAGTT	
miR-6087	TGAGGCGGGGGGGCGAGC	
miR-92a-3p	TATTGCACTTGTCCCGGCCTGT	
miR-93-5p	CAAAGTGCTGTTCGTGCAGGTAG	
RNA5SP226	GTCTACAGCCATACCACCC	CTCCCATCCAAGTACTAACC
RNY4P10	GGTCCGATGGTAGTGGGT	GCCAGTCAAATATAGCAGTGGG
SNORD69	GGATCTGACTGACTGTGCTGA	AACATGAAGCTCAGGGTTGGA
HBB	AGAAGTCTGCCGTTACTGCC	CACCAGCAGCCTGCCC
RNY4P8	GGCTGGTCTGATGGTAGTGGG	GCCAGTCAAATTTAGCAGTGGG
C7orf55	GCCCCAGTGGCCTAATGG	CACCCCAGATGGGACTCG
RNU2-59P	CGGCCTTTTGGCTAAGATC	CTCCTATTCCATCTCCCTGCTC
circ_0000722	CCATCCTCCAGCTTTGCCG	GTGAGGGGGTTGACGGTG
circ_0001305	TCAACAACAAGCTGGAGGATT	GCATATTGGGCATAGCCATC
circ_0001445	TGGGCGAAAGTTCACTTAGAA	CACATGTGTTGCTCCATGTCT

Supplemental Figure Legends

Supp. Figure 1. Sequencing depth and coverage of the *HBB* transcript. Sequencing reads were aligned to the *HBB* transcript. In blue at the bottom is the RefSeq annotation and above are the normalized coverages of reads along each exon in each individual (young and old) of our sequencing cohort (n=23). Read totals are graphed by their density along the entire transcript, with darker bars indicating more coverage of that particular region of each exon or exons.

Supp. Figure 2. Sequencing depth and coverage of the *SNORD69* transcript. Sequencing reads were aligned to the *SNORD69* transcript. In blue at the bottom is the RefSeq annotation and above are the normalized coverages of reads along each exon in each individual (young and old) of our sequencing cohort (n=23). Read totals are plotted by their density along the entire transcript, with darker bars indicating more coverage of that particular region of each exon or exons.

Supp. Figure 3. Sequencing depth and coverage of the *RNY4P8* transcript. Sequencing reads were aligned to the *RNY4P8* transcript. In blue at the bottom is the RefSeq annotation and above are the normalized coverages of reads along each exon in each individual (young and old) of our sequencing cohort (n=23). Read totals are plotted by their density along the entire transcript, with darker bars indicating more coverage of that particular region of each exon or exons.

Supp. Figure 4. Sequencing depth and coverage of the *RNU2-59P* transcript. Sequencing reads were aligned to the *RNU2-59P* transcript. In blue at the bottom is the RefSeq annotation and above are the normalized coverages of reads along each exon in each individual (young and old) of our sequencing cohort (n=23). Read totals are plotted by their density along the entire transcript, with darker bars indicating more coverage of that particular region of each exon or exons.

Supp. Figure 5. Functional interaction analysis of exRNA from young. exRNAs were imported into STRING to formulate a protein-protein interaction network. Each node represents a different exRNA, the colors identify clusters and the lines signify the different functional (direct or indirect) relationship.

Several different clusters are indicated. Line colors represent different types of interactions: turquoise (known from curated databases), pink (known experimentally determined), green (predicted gene neighborhood), red (predicted gene fusions), blue (predicted gene co-occurrence), yellow (textmining), black (co-expression) and purple (protein homology). Dashed lines represent interactions between proteins in different clusters and normal lines are interactions between proteins located within the same cluster.

Supp. Figure 6. Functional interaction analysis of exRNA from old. exRNAs were imported into STRING to formulate a protein-protein interaction network. Each node represents a different exRNA, the colors identify clusters and the lines signify the different functional (direct or indirect) relationship. Several different clusters are indicated. Line colors represent different types of interactions: turquoise (known from curated databases), pink (known experimentally determined), green (predicted gene neighborhood), red (predicted gene fusions), blue (predicted gene co-occurrence), yellow (textmining), black (co-expression) and purple (protein homology). Dashed lines represent interactions between proteins in different clusters and normal lines are interactions between proteins located within the same cluster.

Supp. Figure 7. Validation of circRNAs from serum. RNA was isolated from human PBMCs, HUVECs, or serum and divergent circRNA specific primers were used for RT-qPCR analysis of *circ_000722, circ_001305* and *circ_001445.* Amplification plots and dissociation curves are shown for the serum samples only. RT-qPCR products were visualized by electrophoresis in SYBR-safe (Invitrogen) stained gels. DNA gels were used to visualize that a single amplification product was observed, but since these products were run after the RT-qPCR reaction was complete differences in abundance may not be able to be visualized. Amplification curves were used to calculate relative abundance.

Supp. Figure 8. circRNA changes with human age. RT-qPCR analysis of the indicated circRNAs from the young and old validation cohort. Amplification plots of circRNAs in serum are shown. The RT-qPCR

products from these reactions were visualized by electrophoresis in SYBR-safe (Invitrogen) stained gels. DNA gels were used to visualize that a single amplification product was observed, but since these products were run after the RT-qPCR reaction was complete differences in abundance may not be able to be visualized. Amplification curves were used to calculate relative abundance in Fig. 4F.

Supp. Figure 1. *HBB*

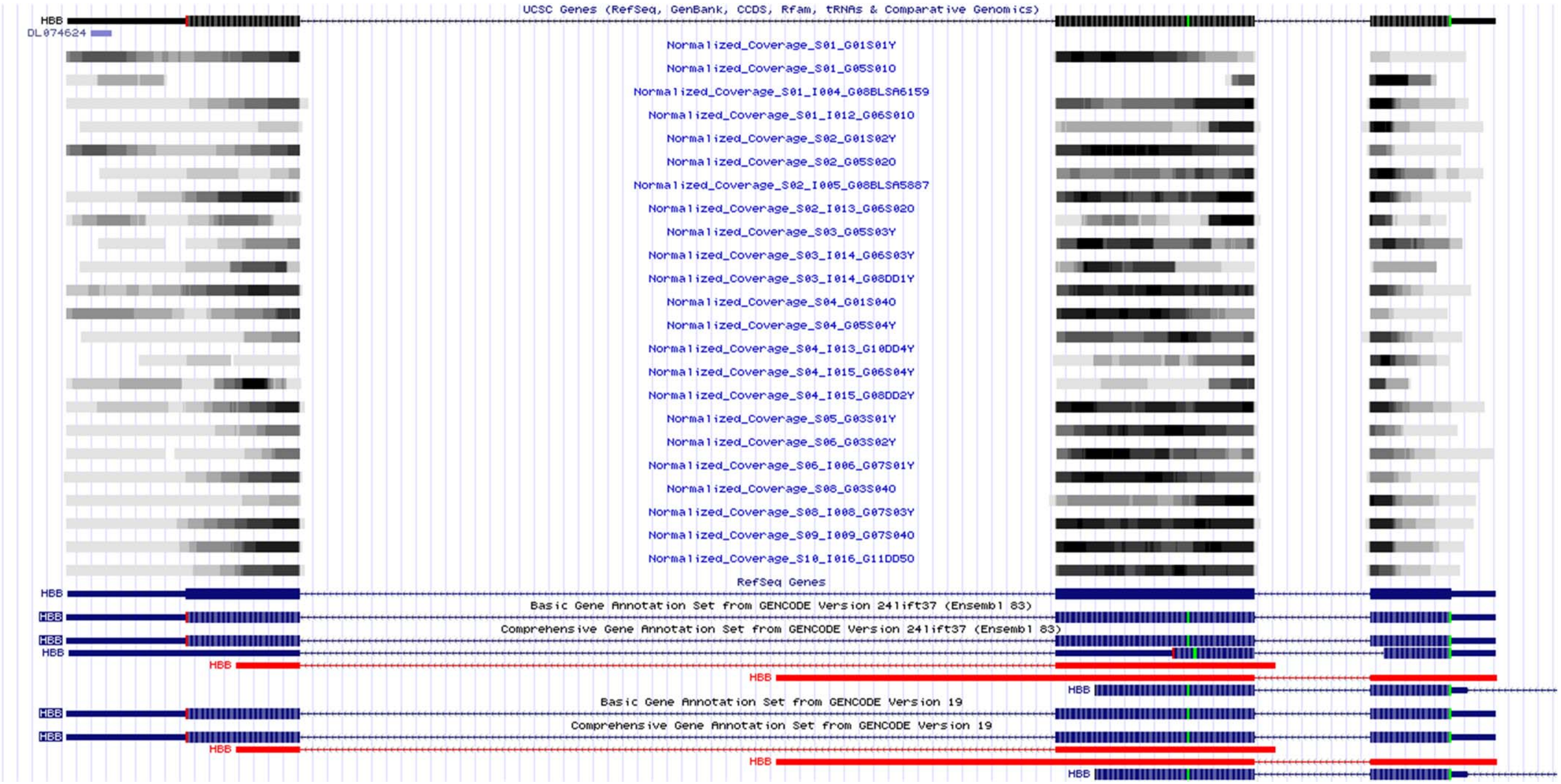

Supp. Figure 2. *SNORD69*

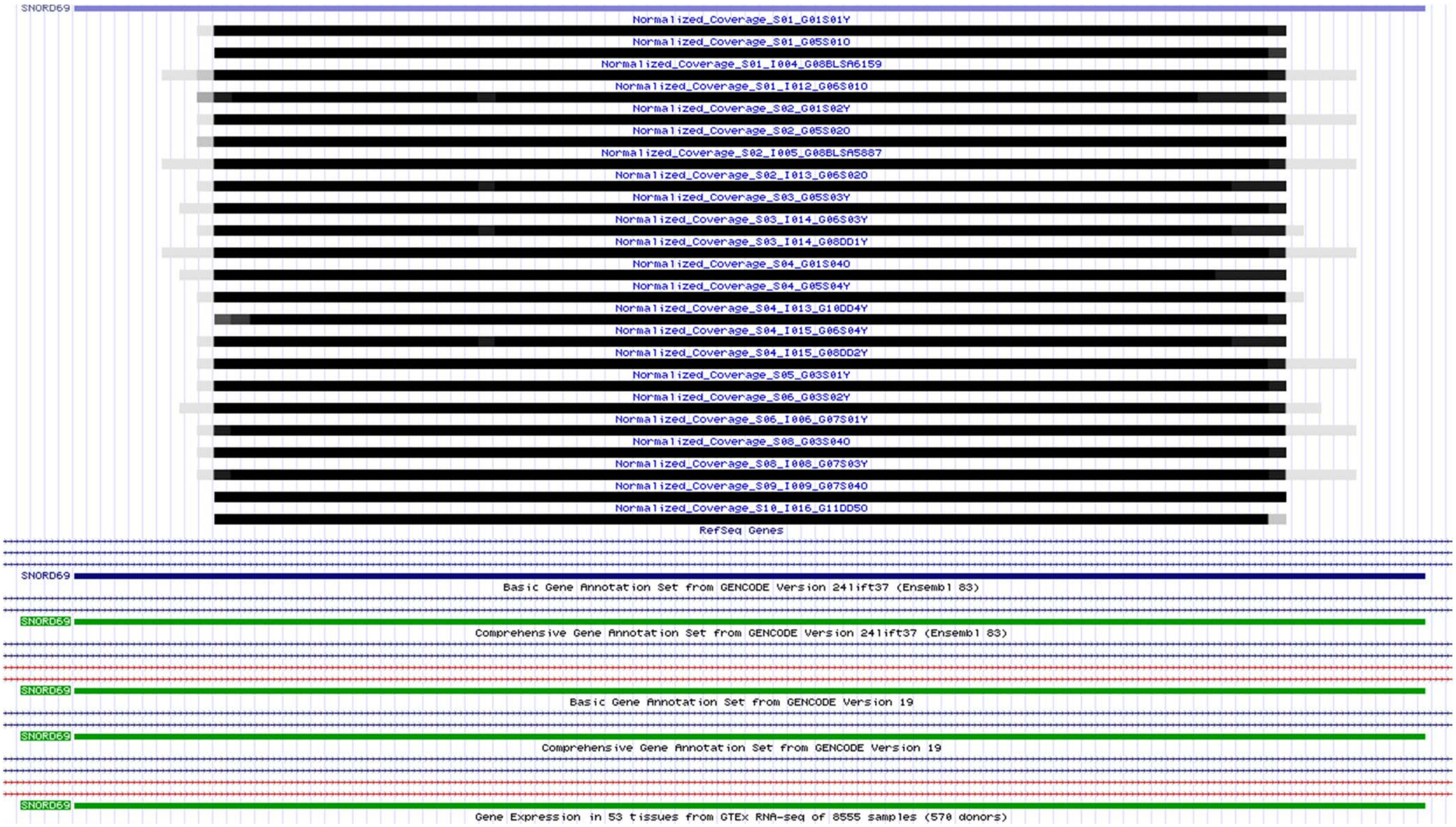

Supp. Figure 3. *RNY4P8*

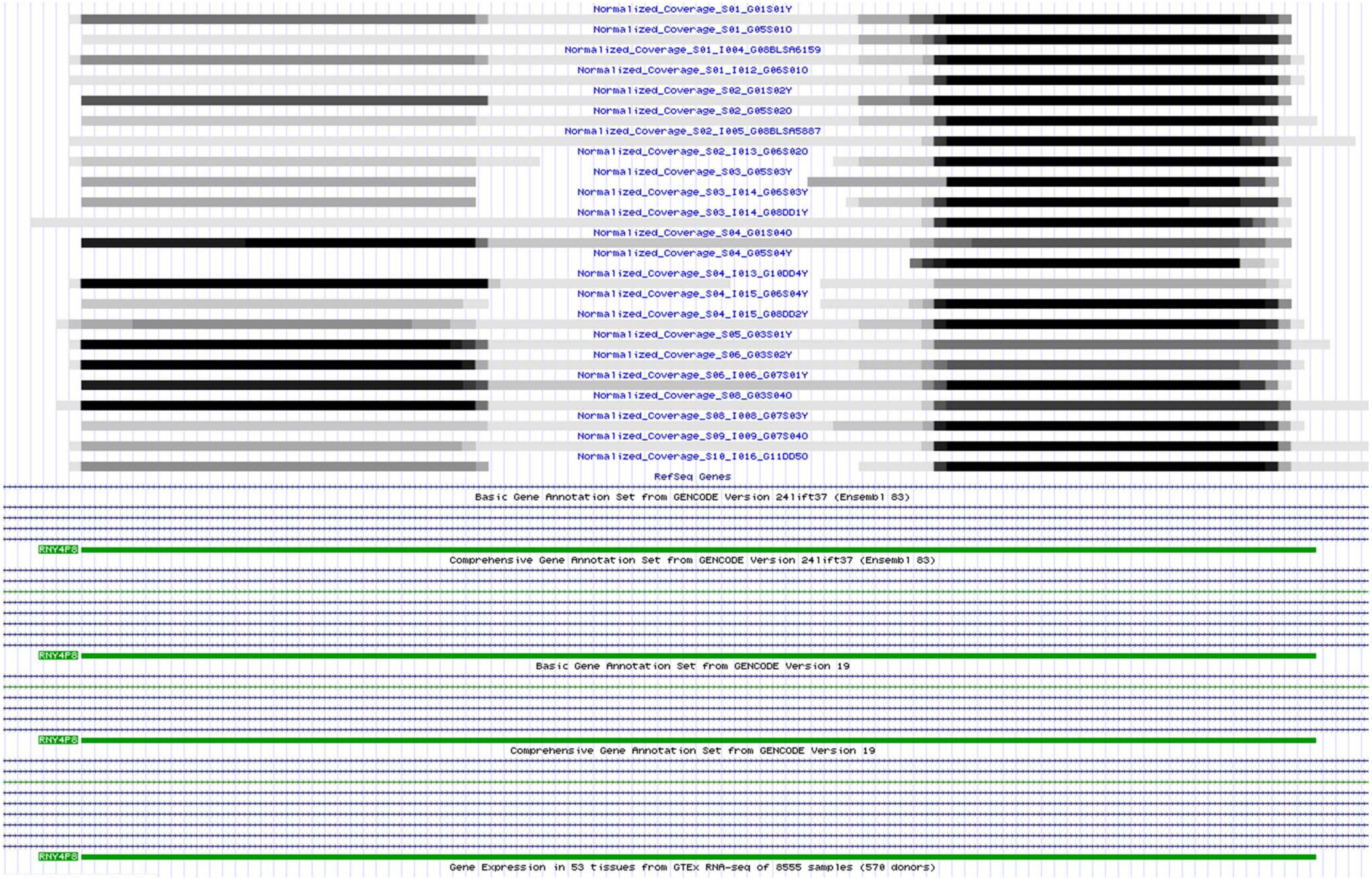

Supp. Figure 4. *RNU2-59P*

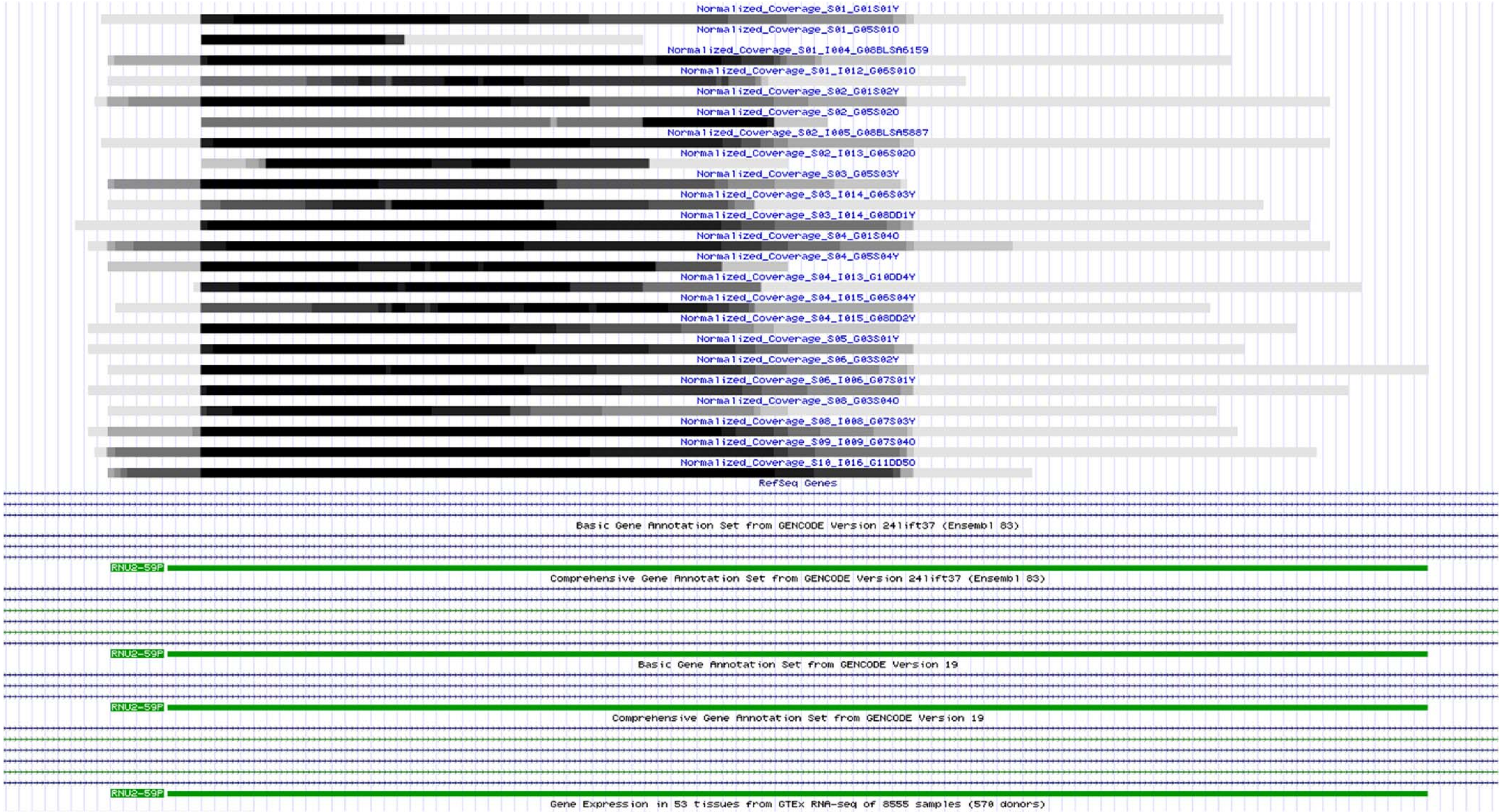

Suppl. Fig. 5

histone RNAs
TF RNAs
other

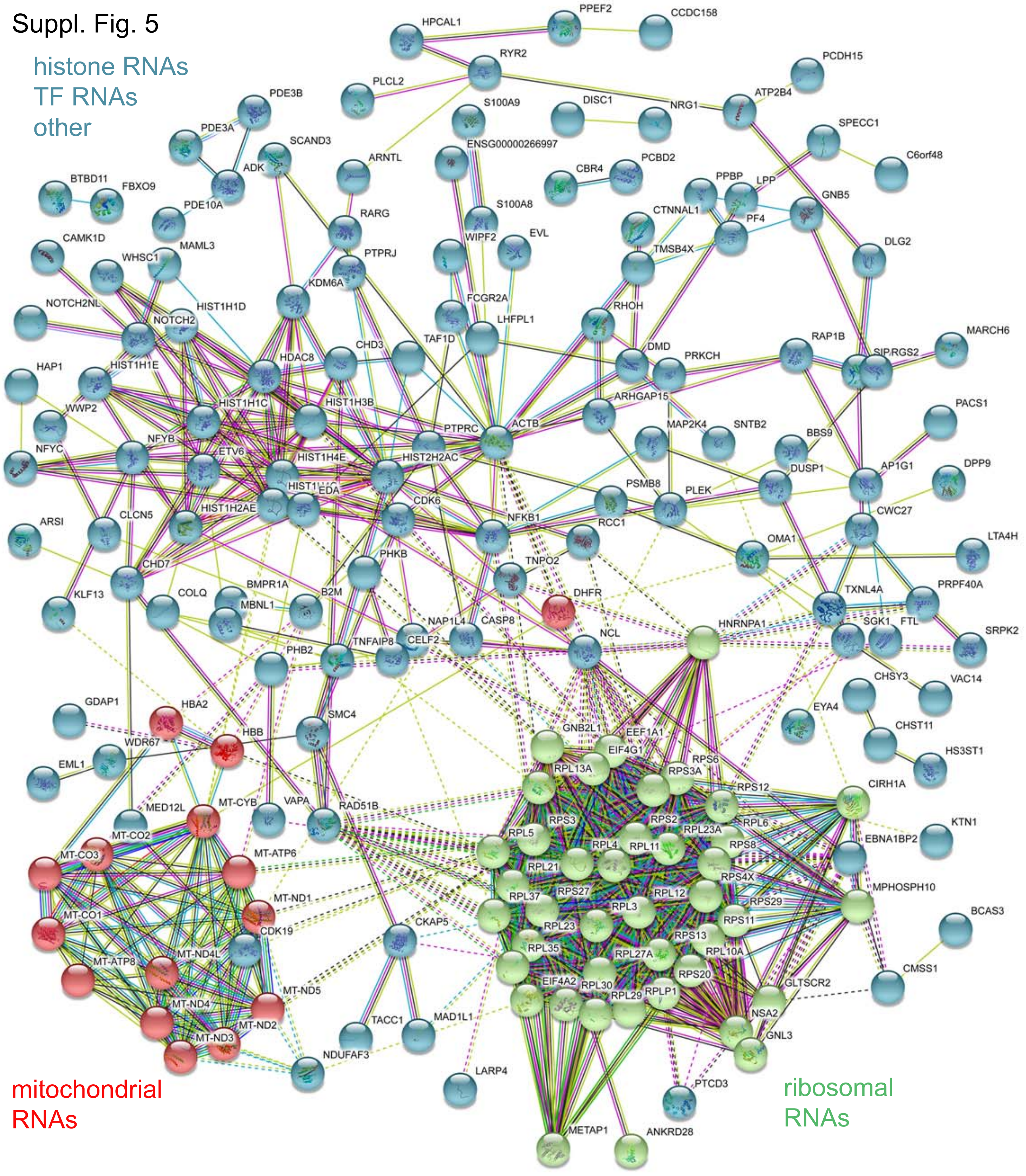

mitochondrial
RNAs

ribosomal
RNAs

Supp. Fig. 6

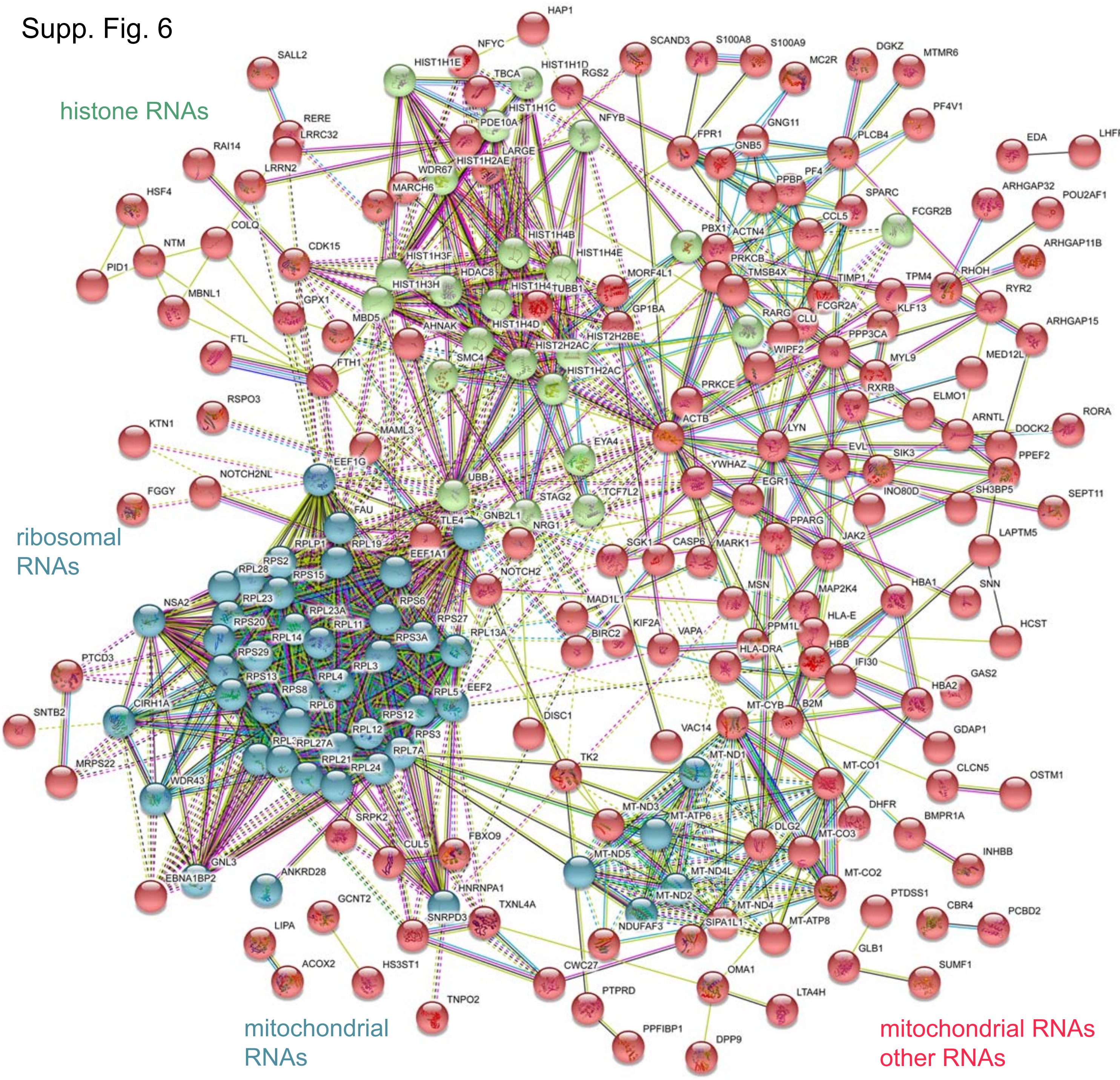
histone RNAs
ribosomal RNAs
mitochondrial RNAs
mitochondrial RNAs
other RNAs

Suppl. Figure 7.

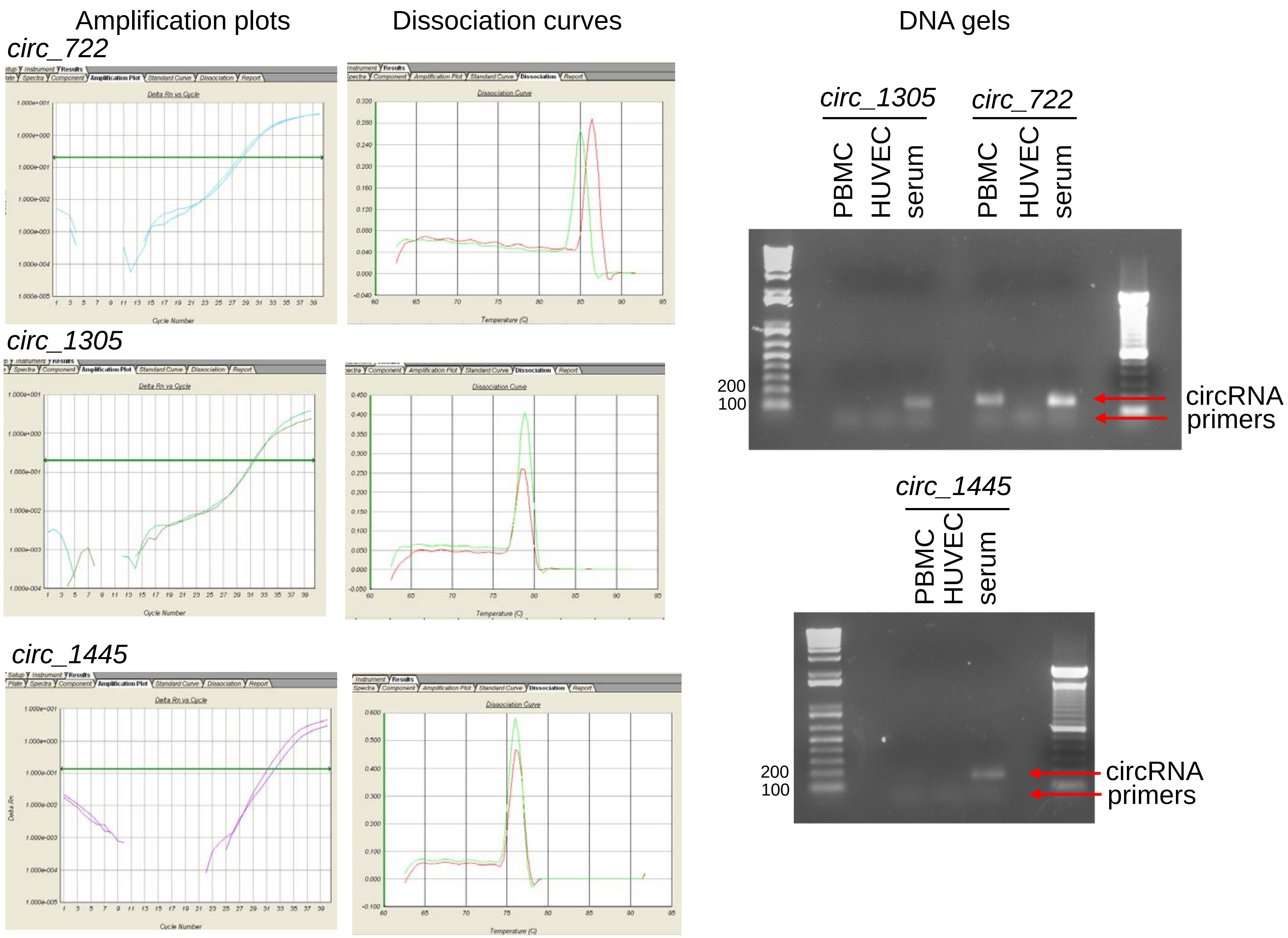

Suppl. Figure 8.

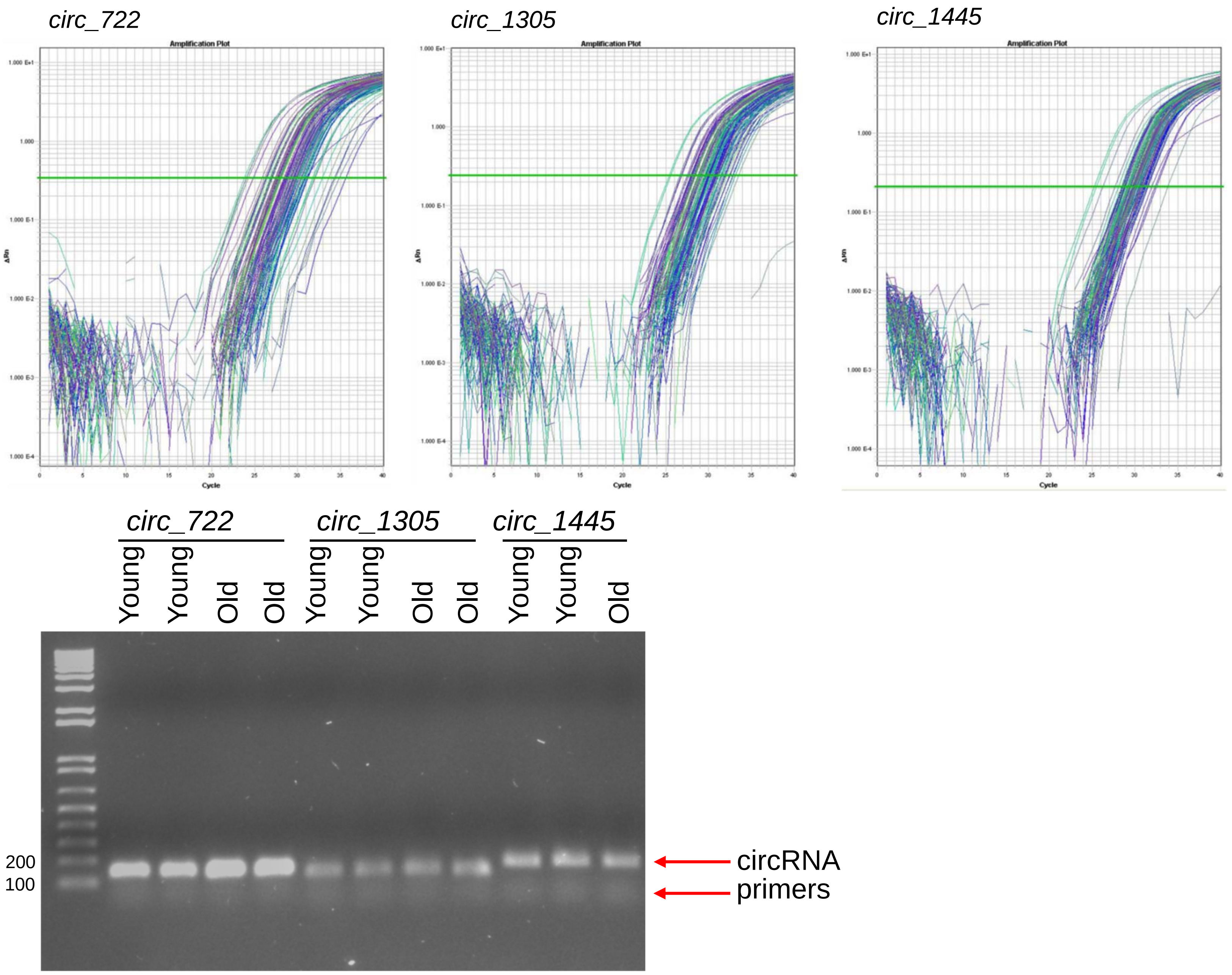

Supplement: Supplementary file 1 [file ACEL-17-na-s001.pdf]
